# Bidirectional phenotypic transition in macrophages influences triple-negative breast cancer cell immune evasive patterns and response to chemotherapies in a co-culture spheroid model

**DOI:** 10.1371/journal.pone.0356198

**Published:** 2026-08-14

**Authors:** Chen Cheng, Brett A. McGregor, Junguk Hur, Colin K. Combs

**Affiliations:** Department of Biomedical Sciences, School of Medicine and Health Sciences, University of North Dakota, Grand Forks, North Dakota, United States of America; Nova Southeastern University, UNITED STATES OF AMERICA

## Abstract

Tumor-associated macrophage (TAM) infiltration is a critical characteristic of triple-negative breast cancer (TNBC) related to drug resistance and poor prognosis. Integrating macrophages into TNBC spheroids is crucial to improve the accuracy of 3D *in vitro* models that mimic the complexity of the tumor microenvironment (TME) and assess treatment response. However, this remains challenging since the reciprocal effects of these two cell types on each other are not fully understood. In this study, we used the TNBC cell line, MDA-MB-231, and polarized M1-like or M2-like macrophages derived from THP-1 monocytes to establish 3D co-culture spheroids to examine bidirectional interactions between these cells and responses to chemotherapy. Drug efficacy, epithelial-mesenchymal transition (EMT) in cancer cells, macrophage phenotypes, and RNA sequencing, including pathway enrichment analysis, were performed in 3D spheroids. CIBERSORTx deconvolution of RNA sequencing results facilitated the separation of cell types within mixtures to estimate their corresponding cell fractions. We observed that M2 macrophages increased the viability of MDA-MB-231 cells in 3D spheroids, while both M1 and M2 macrophages increased the chemosensitivity of 3D spheroids to doxorubicin and paclitaxel. Interestingly, instead of maintaining their phenotypes, both M1 and M2 macrophages lost some polarization and formed a mixed M1-M2 phenotype when co-cultured with MDA-MB-231 cells in 3D spheroids, a phenomenon further supported by RNA-seq deconvolution analysis. However, the fraction of M1-like macrophages shifting to M2-like was much lower than the fraction of M2-like macrophages shifting to M1-like in the 3D co-cultures. Compared with 2D cultures, an expected mesenchymal transition, numerous differentially expressed genes (DEGs) and various pathways, including both tumor-promoting and tumor-suppressing genes, were observed in 3D spheroid MDA-MB-231 cells. However, both M1- and M2-like macrophages induced only partial EMT phenotype changes of cancer cells in co-cultures. Furthermore, a coexistence of pro-inflammatory and anti-inflammatory DEGs was observed in both M1 and M2-like co-cultured cancer spheroids. In conclusion, our findings present an effective 3D co-culture system of breast cancer cells and integrated macrophages for studying dynamic cellular phenotype changes and reciprocal interactions in a heterogeneous environment to mimic aspects of the TME and enhance the accuracy of preclinical *in vitro* treatment response studies.

## Introduction

Breast cancer (BC) is the most commonly diagnosed cancer and the leading cause of cancer malignancy among females worldwide [1]. The World Health Organization (WHO) reported that 2.3 million women were diagnosed with breast cancer, with 670,000 global deaths in 2022 [2]. Triple-negative breast cancer (TNBC) is a highly aggressive subtype of BC characterized by the absence of receptors for estrogen (ER), progesterone (PR), and human epidermal growth factor 2 (HER-2), which limits the efficacy and application of hormone therapies and immunotherapies, leaving conventional therapies as the essential treatment option [3–5]. In addition, the unique tumor microenvironment (TME) of TNBC, including a high level of tumor-infiltrating immune cells, especially tumor-associated macrophages (TAMs), amplifies the complexity of this subtype of breast cancer and contributes to poor prognosis [6].

As the most abundant immune population in the TME, macrophages comprise over 50% of hematopoietic cells and play a pivotal role in cancer cell survival and progression [7]. These macrophages play a dual role in cancer development, with an M1-like pro-inflammatory and immunostimulatory phenotype exhibiting anti-tumor properties, while an M2-like anti-inflammatory, angiogenic, and immunosuppressive phenotype promotes tumor growth and immune evasion [8,9]. In TNBC, M2-like TAMs play predominant roles from tumor initial onset to metastasis, including angiogenesis, invasion, and metastasis promotion, as well as suppression of anti-tumor immune responses and reduction of chemo- and radiotherapy efficacy [10,11]. In addition, studies have demonstrated that TNBC cells possess a strong ability to evade immune clearance via different patterns, such as regulating immune checkpoints, altering epigenetic and metabolic profiles, changing genomic architecture, and recruiting suppressive immune cells, of which the most important ability is to induce macrophages to polarize into an M2-like phenotype, further facilitating tumor cells escape from the immune response [12,13]. Increasing evidence indicates that TAM-targeting therapies, including inhibiting macrophage recruitment, eliminating TAMs, and repolarizing TAMs, have improved therapeutic effects in cancer treatment. Furthermore, a combination of chemotherapies and TAM-targeting therapies has shown promising outcomes in both preclinical and clinical cancer research with a goal of overcoming TAM-mediated resistance [11–14]. Therefore, due to their high plasticity and complex transition abilities, it is essential to precisely understand how tumor cells interact with these macrophages to determine the mechanisms underlying cancer cell growth, invasion, and metastasis, and to identify candidates for targeted therapies [13].

*In vitro* 2D cell culture models have been used for decades in cancer research. However, the complex interactions between various cell types within the TME make it challenging to mimic the *in vivo* tumor environment in 2D cell culture monolayers [14]. Thus, many studies have emphasized the use of 3D cell culture models, which offer unique opportunities to study cell-cell, cell-matrix, and spatial-organizational interactions that influence cellular behavior and responses to therapeutics [15]. Due to these characteristics, 3D tumor cell spheroids are now widely used as an *in vitro* model for investigating various aspects of cancer biology and testing anti-tumor drugs [16]. Given the significant importance of TAMs in tumor development and treatment resistance, it is crucial to include them in the *in vitro* models. Despite attempts to co-culture macrophages and tumor cells in 3D models, challenges still exist in the current co-culture systems, including a lack of standardization, cell population bias, analytical hurdles, and difficulties in multi-cell maintenance. A detailed characterization of the structure and biological characteristics of co-culture spheroids requires further analysis [17,18].

This study aimed to gain better insight into the interaction between polarized macrophages and triple-negative cancer cells in our newly established 3D co-culture spheroids, which mimic aspects of the TME. To achieve this goal, we investigated changes in drug response, cell viability, cell proliferation, epithelial-mesenchymal transition (EMT), and cancer stem cell (CSC) population dynamics in cancer cells when co-cultured with polarized macrophages. We also assessed the phenotype changes of macrophages when they were co-cultured with the cancer cells. In addition, we performed RNA sequencing (RNA-seq) analysis paired with CIBERSORTx deconvolution to identify differentially expressed genes, enriched pathways, and estimate cell type changes in the co-cultured spheroids. To establish a relatively standardized and stable co-culture system and better understand the roles of macrophages in chemoresistance, THP-1-derived macrophages and a more aggressive TNBC cell line, MDA-MB-231, were used in this study. By understanding the macrophage-TNBC axis, combined standard chemotherapies, such as doxorubicin and paclitaxel, with TAM-targeting agents could possibly reprogram the tumor microenvironment and re-sensitize the TNBC cells to the drugs. With these insights, our study may identify new targets for TNBC treatment by considering the complexity of its microenvironment.

## Materials and methods

### Cell culture

The triple-negative breast cancer cell line MDA-MB-231 and the human THP-1 cell line were obtained from ATCC (Manassas, VA, USA). Cells were maintained in D-MEM/F-12 medium (Gibco, Life Technologies, Grand Island, NY, USA) supplemented with 10% fetal bovine serum (FBS) and 1% penicillin/streptomycin (Thermo Fisher Scientific Inc., MA, USA). The cells were incubated at 37 °C in a humidified incubator with 5% CO_2_.

### Macrophage differentiation and polarization

THP-1 cells (passages 5–10) were stimulated with 100 nM phorbol-12-myristate 13-acetate (PMA) (Sigma-Aldrich, Saint Louis, MO, USA) in D-MEM/F-12 medium for 24 hours. Afterward, cells were activated for 24 hours into M1-like macrophages by treatment with 50ng/mL IFN-γ (R&D Systems Inc., Minneapolis, MN, USA) or into M2-like macrophages by treatment with 50ng/mL IL-4 (R&D Systems Inc., Minneapolis, MN, USA).

### 3D Co-cultured spheroids

10,000 MDA-MB-231 cells (passages 9–15) were seeded into each well of a Nunclon Sphera-treated U-shaped-bottom microplate, which is coated with a hydrophilic polymer. (Thermo Fisher Scientific Inc., MA, USA). After 24 h, polarized M1-like or M2-like macrophages were washed three times with sterile PBS to remove polarizing cytokines, and then 5,000 cells were collected and seeded into each well of the microplate containing cancer cells for co-cultures. The co-culture seeding ratio of macrophages to cancer cells (1:2) was determined to better mimic the *in vivo* TME, based on the observation that TAMs typically comprise 10–50% of the TNBC tumor mass [13]. Mono- and co-cultures were incubated for an additional 4 days at 37 °C in a humidified incubator with 5% CO_2_ before analysis or drug treatment (S1 Fig). A 4-day co-culture was selected based on an optimization assay to allow the macrophage infiltration into the spheroids (preliminary data not shown). Spheroid morphology and structure were monitored under an Olympus FV3000 Laser Scanning Confocal Microscope. Spheroids were validated for uniformity based on the relatively well-defined spherical shape and size variation <10% across wells before further experiments were performed.

### Drug treatment and cell viability assay

Doxorubicin (DOX) (CAS# 25316409, Sigma-Aldrich, Saint Louis, MO, USA) and paclitaxel (PTX) (CAS# 33069624, Selleck Chemicals, Houston, TX, USA) were dissolved in DMSO to make stock solutions and diluted with culture media to the desired concentrations for treatment. The final DMSO concentration including the vehicle control was < 0.1% (v/v). Based on our preliminary dose-dependent treatment data in MDA-MB-231 2D and 3D cultures, 50 μM was selected for further analysis to maximize treatment effects (S2 Fig). Mono-culture 3D spheroids of cancer cells and co-culture 3D spheroids of cancer cells and macrophages were treated with 50 μM doxorubicin or paclitaxel for 48 hours. For comparison, MDA-MB-231 cells were seeded into standard 96-well plates and incubated for 5 days to form 2D monolayer cultures. Afterward, 2D cultured cells were treated with 50 μM doxorubicin or paclitaxel for 48 hours. Additional comparisons in MDA-MB-231 and M1/M2 macrophages 2D co-cultures were also performed for reference (S2 Fig).

The viability of 2D- and 3D-cultured cells was measured using WST-1 (Sigma-Aldrich, Saint Louis, MO, USA) according to the manufacturer’s instructions. Cells without treatment were used as media controls. Briefly, 20 μL of WST-1 reagent was added to each well. The plates were incubated at 37°C for 4 hours, and the absorbance was measured at 450 nm using the ELx800 plate reader (Bio-Tek Instruments, Winooski, VT, USA). To prevent chemical intrinsic absorbance from interfering with the assay results, background absorbance was corrected by including cell-free blanks (wells containing corresponding chemicals and WST reagent without cells). Experiments were performed in seven biological replicates.

### Fluorescence imaging

Fluorescent images of cells were recorded by confocal laser scanning microscopy (CLSM). 3D mono-cultured and co-cultured cell spheroids were formed and treated as described above. MDA-MB-231 cells were seeded in a 4-chamber glass-bottom cell culture dish (Cellvis, Mountain View, CA, USA) at a density of 2.5 × 10^5^ cells/well and treated with 50 μM doxorubicin or paclitaxel for 48 hours for comparison. After treatment, the viability and morphology of the cells were assessed using the Cyto3D™ Live-Dead Assay Kit (TheWell Bioscience, Monmouth Junction, NJ, USA) according to the manufacturer’s instructions. 2 µL of Cyto3D reagent was added to every 100 µL of media in each well. Cells were cultured at 37°C for 10 minutes and then imaged under a confocal microscope (Olympus FV3000 Laser Scanning Confocal Microscope). The live cells were excited at (Ex/Em) 494/517 nm, while the dead cells were excited at (Ex/Em) 535/617 nm. Doxorubicin was not included due to its intrinsic fluorescence at (Ex/Em) 480/595 nm, which can overlap with the dead-cell marker. To confirm the distribution of different types of cells in the 3D spheroids, MDA-MB-231 cells (CellTracker Green CMFDA, Thermo Fisher Scientific), M1 macrophages (CellTracker Red CMTPX, Thermo Fisher Scientific), and M2 macrophages (CellTracker Deep Red, Thermo Fisher Scientific) were stained according to the manufacturer's instructions before seeding to form 3D spheroids. Cells were observed under the confocal microscope after co-culturing.

To evaluate drug penetration into the 3D spheroids, 50 μM doxorubicin and Oregon Green 488-conjugated paclitaxel were used to treat mono-cultured and co-cultured cells for 48 hours. After treatment, the cells were washed three times with PBS and then fixed with 4% paraformaldehyde for 10 minutes. Nuclear DNA was stained with Hoechst 33342 (Thermo Fisher Scientific Inc., MA, USA). After three additional washes with PBS, the cells were imaged using an Olympus confocal microscope. All spheroids were transferred to glass-bottom cell culture dishes using cut-edge pipette tips before imaging under a confocal microscope. Experiments were performed in three biological replicates.

### Flow cytometry

Phenotypic changes in MDA-MB-231 cells and macrophages in mono-cultured and co-cultured spheroids were assessed by multicolor flow cytometry (BD FACS Symphony A3). 2D and 3D cell samples were dissociated using TrypLE Express Enzyme (1x) (Thermo Fisher Scientific Inc., MA, USA), neutralized with serum-supplemented media, and centrifuged for 5 min at 1500 rpm. After washing with PBS (Ca2 + -/ Mg2 + - free), cells were harvested and resuspended in viability dye (Ghost dye 510, 1:1000; Tonbo Biosciences, CA, USA) prepared in PBS (Ca2 + -/ Mg2 + - free). After incubation for 30 minutes at 4°C, the cells were centrifuged and washed with FACS buffer (Ca2 + - and Mg2 + -free PBS supplemented with 2% fetal bovine serum). Then, the cells were incubated with Human TruStain FcX-Fc Receptor Blocking Solution (BioLegend, CA, USA) at a 1:50 dilution for 10 minutes on ice. Cells were stained with anti-human antibodies against CD45 (HI30), CD24 (IT2.2), CD44 (BJ18), CD324 (67A4), laminin (Polyclonal), fibronectin (1G10F9), vimentin (O91D3), CD325 (8C11), α-SMA (1A4/asm-1), β Catenin 1(15B8), FSP1 (NJ-4F3-D1), CD120b (3G7A02), CD284 (HTA125), CD16 (3G8), CD209 (9E9A8), CD206 (MMR 15−2), CD38 (HIT2), CD86 (ML5), CD14 (63D3), CD319 (162.1), CD80 (2D10), CD163 (GHI/61), CD68 (Y1/82A), CD32 (FUN-2), CD93 (VIMD2). Laminin and α-SMA antibodies were purchased from Bio-Techne, USA. Fibronectin antibody was purchased from Proteintech, USA. All other antibodies used in this study were purchased from BioLegend, USA. Detailed information on antibodies is summarized in S1 Table.

For proliferation analysis, Tag-it Violet™ Proliferation and Cell Tracking Dye (BioLegend, CA, USA) was used according to the manufacturer’s instructions. Briefly, cells were incubated with dye for 20 minutes at 37°C and kept in the dark. After washing with pre-warmed media, cells were ready for downstream applications or analysis. Doublet exclusion was performed using FSC-A versus FSC-H to isolate single cells, and a viability dye was utilized to exclude dead cells as described above. Viable cells were identified as Ghost Dye negative, and dead cells were identified as Ghost Dye positive. Single-stained, unstained, and fluorescence one minus (FMO) controls were used to set compensation and manual gating, ensuring that shifts in autofluorescence or cell granularity did not affect the specificity of the gating. To account for potential variations in macrophage activation, the gating was anchored on specific phenotypic marker CD45 rather than scatter properties alone. Macrophages were identified as the CD45 + leukocyte population, whereas cancer cells were gated as the CD45- fraction. The percentage of viable cells within the target population was calculated using the following formula: Cell Viability (%) = (Ghost Dye negative cells/ Total number of cells) × 100. Data were collected on a BD FACS Symphony A3 and analyzed using FlowJo software (BD Life Sciences, Ashland, OR) (S3 Fig). Experiments were performed in three biological replicates.

### RNA extraction and sequencing

Cells were washed in PBS and lysed using RNAzol RT according to the RNAzol® RT Column Kit (Molecular Research Center, Cincinnati, OH) protocol. Briefly, the lysate was mixed with water and centrifuged at 12,000 × g to remove DNA and proteins. The lysate was then diluted at a 1:1 ratio with isopropanol and added to a column, followed by another 12,000 × g centrifugation. RNA was then washed twice with 100% ethanol to remove impurities. RNA was then eluted using RNase-free deionized water and stored at −80°C. Concentrations were measured using a Qubit 4 Fluorometer, and purity was checked using a NanoDrop One spectrophotometer (Thermo Fisher Scientific, Waltham, MA, USA). Prepared samples were indexed, pooled, and sequenced on an Illumina NovaSeq 6000. Subsequent basecalling and demultiplexing were performed according to standard bioinformatic pipelines.

FASTQ files were obtained from RNA sequencing and evaluated using FastQC (version 0.11.9) for quality assessment [19]. Raw reads were trimmed using Trimmomatic (version 0.39) [20]. HISAT2 (version 2.2.1) was used to map cleaned reads to the hg38 human reference genome, and the Rsubread (version 2.18.0) featureCounts function assigned successfully mapped reads to unique genomic features [21,22]. Differentially expressed genes (DEGs) were identified using DESeq2 (version 1.44.0) [23], applying a significance threshold of Benjamini-Hochberg adjusted p-value <0.05. Overall DEGs from each comparison are demonstrated by a volcano plot using EnhancedVolcano (version 1.24.0) [24,25]. Experiments were performed in four biological replicates.

### Deconvolution analysis

Bulk sequencing results from co-cultured cells were processed using CIBERSORTx for deconvolution analysis using single-cell type cultures as a sorted reference [26]. Cell fractions were estimated using the fractions function from CIBERSORTx across 100 permutations with sample Fragments Per Kilobase of transcript per Million mapped reads (FPKMs) as the submitted mixture and FPKM from single-cell type culture samples as the reference. FPKM files, as well as obtained cell fraction results, were processed by the CIBERSORTx gep function (gene expression profile) to impute each cell type's group expression profiles across all samples. Filtered gep results were then submitted to the hires function for high-resolution sample-level imputed gene expression of distinct cell types from each sample. Imputed gene counts from CIBERSORTx hires function using M1 polarized macrophages, M2 polarized macrophages, MDA-MB-231 (2D), and MDA-MB-231 (3D) cell types were also processed for DEGs using DESeq2 with a significance threshold of <0.05 Benjamini-Hochberg adjusted p-value [23]. Following imputation, overall DEGs were displayed by a volcano plot using EnhancedVolcano (version 1.24.0) [24].

### Functional enrichment analysis

Significant DEGs from both bulk analysis and high-resolution deconvolution and imputation were subject to enrichment analysis using our in-house R package richR (https://github.com/hurlab/richR) to determine enriched functional terms represented by identified DEGs. For each comparison, genes with a Benjamini-Hochberg adjusted p-value <0.05 were submitted for enrichment analysis. Functional terms and pathways from Gene Ontology (GO), Kyoto Encyclopedia of Genes and Genomes (KEGG), and Reactome Pathway Database were used to query the DEG lists, and significant terms were determined using a cutoff of <0.05 Benjamini-Hochberg adjusted p-value [27–34]. Enrichment results were visualized as a dot plot using RichR, with dot size indicating the number of genes within our data annotated to each function and dot color representing significance using a -log10(adj p-value) scale. GO, KEGG, and Reactome results were clustered based on overlapping genes between functions to reduce redundant terms [35].

### Cytokine assessment

RayBio Quantibody® Human Cytokine Array 1 (QAH-CYT-1, RayBio, Norcross, GA) was used to assay medium from MDA-MB-231 2D cultures, 3D monocultures, and co-cultures with M1/M2 macrophages, according to the manufacturer's instructions. Twenty different cytokines were evaluated: GM-CSF, GRO alpha/beta/gamma, IFN-gamma, IL-1 alpha, IL-1 beta, IL-10, IL-12 p70, IL-13, IL-2, IL-4, IL-5, IL-6, IL-8 (CXCL8), MCP-1 (CCL2), MIP-1 alpha (CCL3), MIP-1 beta (CCL4), MMP-9, RANTES (CCL5), TNF-alpha, VEGF-A. RayBioTech used a GenePix 4400 scanner to scan the slide arrays with GenePix Pro software. Results were analyzed using the RayBio Analysis Tool. Using a standard curve, concentrations of each cytokine were determined (pg/mL).

### Statistical analysis

Results are presented as mean ± SD from at least three independent experiments unless otherwise indicated. Data with more than two groups were analyzed statistically by one-way analysis of variance followed by Tukey’s multiple comparisons test for comparisons across all experimental groups or Šídák's multiple comparisons test for pre-planned pairwise comparisons. A two-tailed unpaired t-test was used for comparison between the two groups. For RNA-seq analysis, gene-level raw count data were analyzed using DESeq2 in R. Culture group was modeled as the experimental factor, with four biological replicates included per group. Differential expression was assessed using the default DESeq2 workflow, including size-factor normalization, dispersion estimation, and negative binomial generalized linear modeling. Pre-specified pairwise contrasts between culture groups were evaluated using the DESeq2 results function. P-values were adjusted for multiple testing using the Benjamini-Hochberg false discovery rate method, and genes with an adjusted p-value <0.05 were considered differentially expressed.

## Results

### Co-culture with M2-like macrophages increased the viability of MDA-MB-231 cells in 3D spheroids

**Fig 1:**
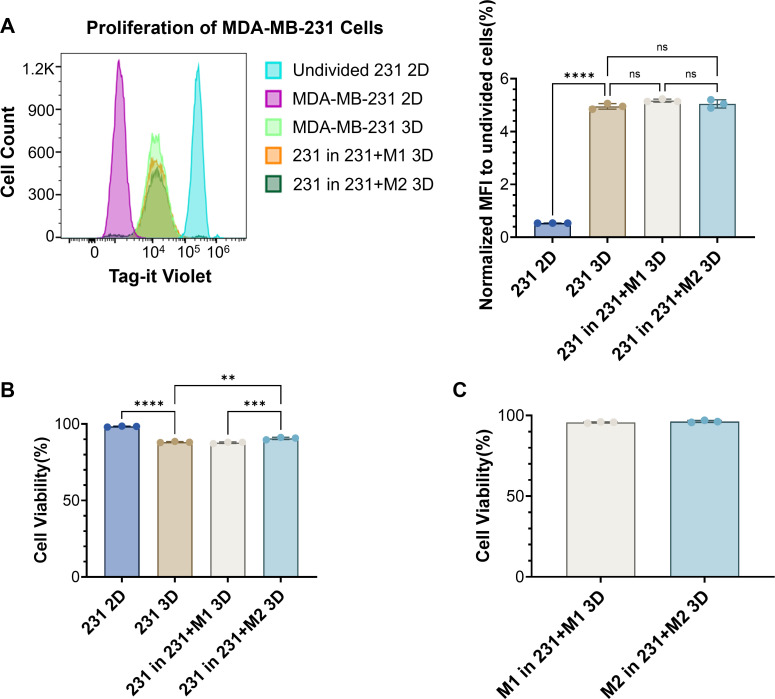
Viability and proliferation of MDA-MB-231 cells and M1/M2-like macrophages in 2D monolayer culture and 3D spheroids by flow cytometry analysis. (A) MDA-MB-231 cell proliferation in 2D monolayer, 3D mono-cultured, and co-cultured spheroids. Tag-it Violet-labeled MDA-MB-231 cells were mono-cultured or co-cultured with M1 or M2-like macrophages for 4 days and assessed by flow cytometry. Median fluorescence intensity (MFI) was compared with undivided MDA-MB-231 2D cells. (B) MDA-MB-231 cell viability in 2D monolayer, 3D mono-cultured, and co-cultured spheroids. Cells were stained with ghost dye violet 510 and assessed by flow cytometry. (C) Macrophage viability in co-cultured spheroids. Results are presented as mean ± SD; n = 3, p* < 0.05, p** < 0.01, p*** < 0.001, p**** < 0.0001.

To establish the 3D co-cultured spheroids, MDA-MB-231 cells were plated into Nunclon Sphera-treated U-shaped-bottom 96-well microplates. Polarized M1- or M2-like macrophages from stimulated THP-1 cells were added after 24 hours and co-cultured with the MDA-MB-231 cells for 4 days before further treatment or analysis. Co-existence of cancer cells (CD45-) and macrophages (CD45+) in the spheroids was confirmed by flow cytometry and confocal microscopy, which showed both M1 and M2 macrophages effectively infiltrated in the co-culture spheroids (S3 and S4 Figs). Ghost dye Violet 510 was used to assess the viability of M1/M2-like macrophages and MDA-MB-231 cells in 2D and 3D cultures by flow cytometry, while Tag-it Violet was used to determine the proliferation rate of cancer cells under different culture conditions through mean fluorescence intensity (MFI) assessment. The undivided peak of tag-it violet proliferation analysis was defined using the fully-stained 2D MDA-MB-231 cell controls (generation 0). MDA-MB-231 cells in 3D mono-cultured spheroids were compared with cancer cells in 2D culture and in 3D co-cultures. As displayed in Fig 1A and 1B, MDA-MB-231 cells in 3D mono-cultured spheroids showed a slower proliferation rate as well as a lower cell viability compared to the 2D monolayer cultures. M2-like polarized macrophages significantly increased the viability but not the proliferation rate of cancer cells in the co-cultured 3D spheroids, while no significant cell viability difference was observed between M1- and M2-like polarized macrophages in the co-cultured 3D spheroids (Fig 1C and S2 Table), suggesting that M2-like macrophages affected MDA-MB-231 cell viability in the 3D co-cultures.

### Co-culture with either M1- or M2-like macrophages increases the chemotherapy response of 3D spheroids

**Fig 2:**
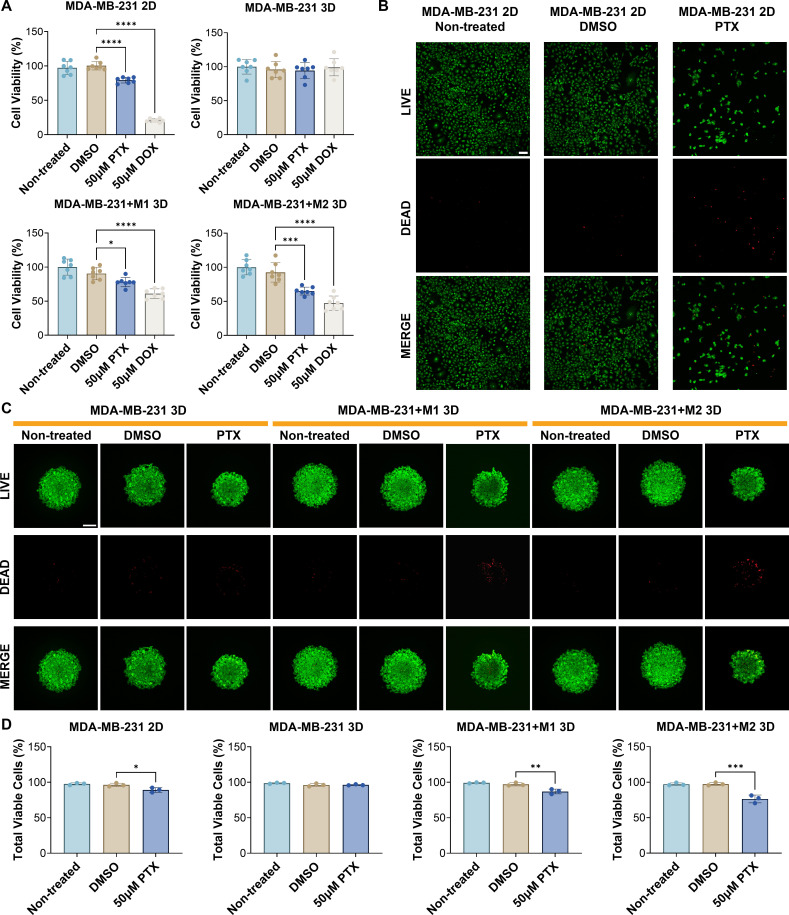
Treatment response of 2D monolayer MDA-MB-231 cells and 3D mono-cultured/co-cultured spheroids. Cells were exposed to DMSO, 50 μM paclitaxel, or 50 μM doxorubicin for 48 hours of treatment. (A) WST assays were performed as an indicator of cell viability. (B) 2D monolayer cultured cell survival was examined using a live (green)/dead (red) staining assay. Scale Bar = 100μm. (C) 3D mono-cultured and co-cultured cells were stained with a live/dead kit. Scale Bar = 500μm. Images were taken at ~170 µm from the bottom of spheroids. (D) Quantification of the live/dead cells staining. Results from the treated groups were compared with the vehicle group. Results are presented as mean ± SD; n = 7, p* < 0.05, p** < 0.01, p*** < 0.001, p**** < 0.0001.

Paclitaxel and Doxorubicin were added to MDA-MB-231 2D monolayer cultures, 3D mono-cultured spheroids, and 3D co-cultured spheroids with M1- or M2-like macrophages for 48h to compare chemosensitivity responses. Compared with 2D monolayer cultures, MDA-MB-231 cells showed treatment resistance to both PTX and DOX in the 3D mono-cultured spheroids. However, when co-cultured with either M1- or M2-like macrophages in 3D spheroids, viability of the treated cells significantly decreased, indicating an increasing drug sensitivity in the 3D spheroids (Fig 2A). Confocal fluorescence images of the 2D monolayer cultures showed a dramatic increase in dead MDA-MB-231 cells along with an obvious morphology change in the cells after the 48h treatment of PTX (Fig 2B). Compact and round-shaped structures with a relatively well-defined outer perimeter were observed in both 3D mono-cultured and co-cultured spheroids without treatment (Fig 2C). Both mono-cultured and co-cultured spheroids showed relatively intact organization after PTX treatment, with an observed smaller shape compared with their respective control. A robust increase in dead-cell staining was observed in the PTX-treated spheroids of both M1- and M2-like macrophage co-cultures with the cancer cells (Fig 2C). These results indicated that the drug response pattern would alternate in 3D cancer cell spheroids when co-culturing with both M1- and M2-like macrophages.

**Fig 3:**
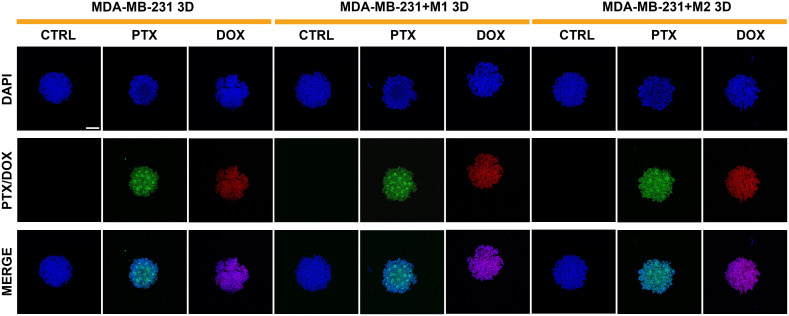
Paclitaxel (PTX) and Doxorubicin (DOX) penetration into 3D cultured spheroids. MDA-MB-231 3D mono-cultured or co-cultured spheroids with M1/M2-like macrophages were exposed to DMSO, 50 μM pre-labeled paclitaxel, or doxorubicin for 48 hours. Drug fluorescence was visualized and compared to Hoechst labeling of cells. Scale Bar = 500μm.

To assess whether limited penetration of PTX/DOX into the spheroids might be responsible for the increased chemoresistance in 3D spheroids, we treated spheroids with pre-labeled AF488 PTX or DOX for 48h to assess penetration of the drugs into the mono- and co-cultured spheroids. As shown in Fig 3, both PTX and DOX were able to penetrate the cores of all spheroid cultures, suggesting that the chemoresistance response of 3D spheroids versus 2D monolayer cultures was not due to inability of the drugs to infiltrate the spheroids.

### Co-culture with M1/M2-like macrophages regulated CD44/CD24 subpopulations and partially attenuated the epithelial-mesenchymal transition (EMT) of MDA-MB-231 cells in 3D spheroids

**Fig 4:**
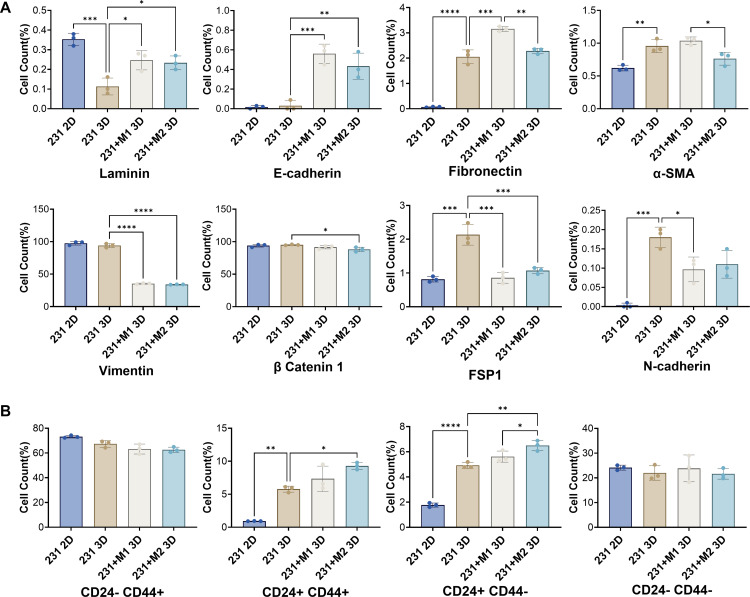
Phenotype changes of MDA-MB-231 cells in 2D monolayer culture, 3D mono-cultured, and co-cultured spheroids with M1/M2-like macrophage cells. (A) Flow cytometry analysis of epithelial and mesenchymal marker levels in MDA-MB-231 cells (CD45-). Laminin, E-cadherin, Fibronectin, α-SMA, FSP1, N-cadherin, Vimentin, and β-Catenin 1 expressions were analyzed by flow cytometry. (B) Flow cytometry analysis of CD24 and CD44 levels in MDA-MB-231 cells. Results are presented as mean ± SD; n = 3, p* < 0.05, p** < 0.01, p*** < 0.001, p**** < 0.0001.

To explore whether the 3D MDA-MB-231 spheroids had an altered progression of epithelial to mesenchymal transition (EMT) compared to 2D cultures and to assess the effect of macrophages on EMT, epithelial markers, laminin and E-cadherin, and mesenchymal markers, fibronectin, α-SMA, vimentin, β-catenin 1, FSP1, and N-cadherin were analyzed by flow cytometry after 3D mono-culture or co-culture with either M1- or M2-like macrophages. Cancer cells (CD45-) and macrophages (CD45+) were separated in co-cultured spheroids before sequential analysis. MDA-MB-231 cells in 3D mono-cultured spheroids were compared with cancer cells in 2D culture and in 3D co-cultures. As expected, compared to the 2D monolayer cultures, downregulated laminin as well as upregulated fibronectin, α-SMA, FSP1, and N-cadherin levels were observed in 3D mono-cultured spheroids (Figs 4A and S5). Although no obvious difference was found in the percentage of E-cadherin, vimentin and β-catenin 1 cell counts, MFI results showed significantly increased E-cadherin and vimentin, along with decreased β-catenin 1 in 3D monocultures compared with 2D culture. Interestingly, co-culture with both M1- and M2-like macrophages increased laminin and E-cadherin levels, but decreased vimentin, FSP1 and N-cadherin levels in 3D MDA-MB-231 cells (Figs 4A and S5). Compared with the MDA-MB-231 3D mono-cultures, increased fibronectin levels were only observed in M1-like macrophage co-culture spheroids, whereas decreased β-catenin 1 levels were only found in M2-like macrophage co-culture spheroids (Figs 4A and S5).

To investigate the stem cell properties of the 3D TNBC cancer cell spheroids and the effects of macrophages on these markers, we next compared the expression of CD44 and CD24 in MDA-MB-231 cells using flow cytometry analysis. CD24^-^CD44^+^ is considered a stem-like marker highly related to the malignancy of breast cancer [36]. As expected, high CD44^+^CD24^-^ expression was observed in MDA-MB-231 cells within all groups. Interestingly, both CD24^+^CD44^+^ and CD24^+^CD44^-^ subpopulations increased in 3D MDA-MB-231 spheroids when compared with 2D cultures, with a significantly higher expression when co-culturing with M2-like macrophages (Figs 4B and S5). These results indicated that co-culture with both M1- and M2-like macrophages attenuated the EMT process of 3D MDA-MB-231 cells, while only M2-macrophages modulated the CD44/CD24 subpopulation in the co-cultures.

### Transitions between M1- and M2-like macrophages when co-cultured with MDA-MB-231 cells in 3D spheroids

**Fig 5:**
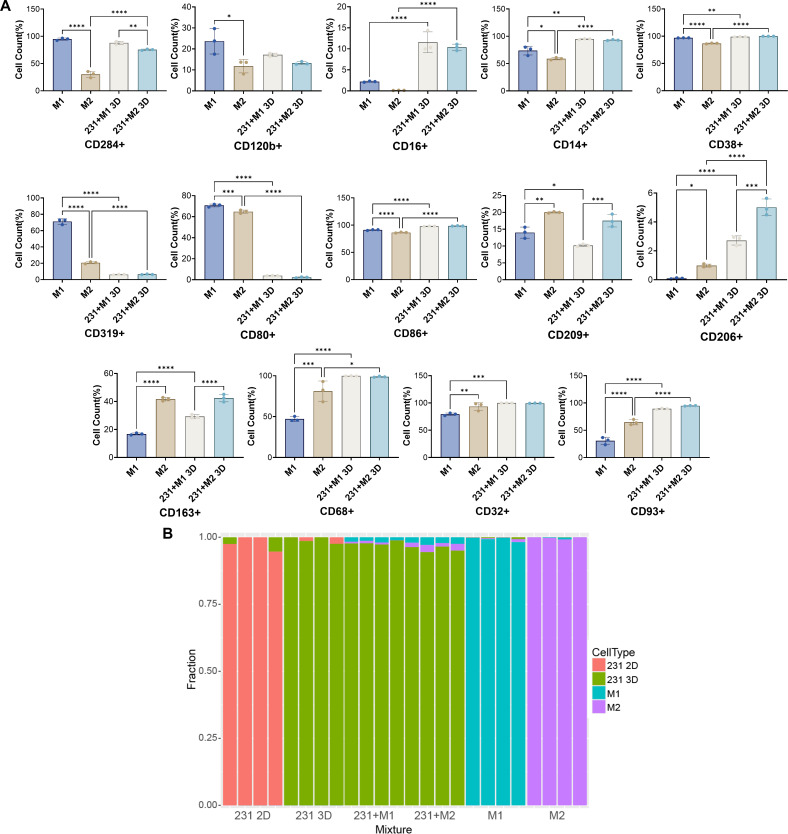
Characterization of M1/M2-like macrophages in 2D monolayer culture and 3D co-cultured spheroids with MDA-MB-231 cells. M1 and M2-like macrophages were collected after polarization or after co-culturing with MDA-MB-231 cells in 3D spheroids. (A) Flow cytometry analysis of CD45 + M1/M2-like macrophage marker levels in 2D monolayer and 3D co-cultured spheroids. CD284, CD120b, CD16, CD14, CD38, CD319, CD80, CD86, CD209, CD206, CD163, CD68, CD32 and CD93 were analyzed. Results are presented as mean ± SD; n = 3, p* < 0.05, p** < 0.01, p*** < 0.001, p**** < 0.0001. (B) Cell fraction analysis of 2D MDA-MB-231 cells, 3D MDA-MB-231 mono-cultured spheroids, polarized M1/M2-like macrophages, and 3D co-cultured spheroids of MDA-MB-231 cells and M1/M2-like macrophages was performed by deconvolution analysis of bulk RNA sequencing data. Each color indicates the proportion of total sequences attributed to each cell type after deconvolution. Each bar represents an individual cell sample included in the RNA deconvolution analysis (n = 4).

To characterize the differences in macrophage polarization under the influence of TNBC cancer cells in 3D cultured spheroids, co-cultured M1/M2 macrophages and MDA-MB-231 3D spheroids were collected after 4 days of culturing for flow cytometry analysis. Separate polarized M1 and M2 macrophages were collected for comparison. The expression of typical macrophage cell-surface markers was analyzed by flow cytometry. As expected, polarized M1-like macrophages expressed higher levels of CD284, CD120b, CD16, CD14, CD38, CD319, CD80 and CD86, while M2-like macrophages expressed higher levels of CD209, CD206, CD163, CD68, CD32 and CD93 (Figs 5A, S6 Fig and S3 Table). After 4 days of co-culture with MDA-MB-231 cells in 3D spheroids, CD284 remained at high levels in co-cultured M1-like macrophages while CD209, CD206, and CD163 remained at high levels in the co-cultured M2-like macrophages. Interestingly, levels of CD120b, CD14, CD38, CD319, CD80, CD86, CD68, CD32 and CD93 lost their significant differences between co-cultured M1- and M2-like macrophages (Fig 5A, S6 Fig and S3 Table).

To better characterize the phenotype changes of the macrophages, we next sequenced the whole transcriptome of both MDA-MB-231 cells and macrophages in the 3D co-cultured spheroids and compared their gene expression profiles to those of individually cultured cells. Bulk RNA sequencing results from co-cultured cells were processed to separate different cell types using CIBERSORTx for deconvolution analysis. As shown in Fig 5B, CIBERSORTx revealed a dramatic change in macrophage cell fractions between M1- and M2-like cells in 3D co-cultured spheroids. Interestingly, the fraction of M1-like macrophages shifting to M2 was much lower than the fraction of M2-like macrophages shifting to M1 in the 3D co-cultures. Combined with flow cytometry analysis, our findings indicated that, rather than maintaining their phenotypes, M1- and M2-like macrophages lost some polarization and formed a mixed M1/M2-like phenotype when co-cultured with MDA-MB-231 cells, suggesting high phenotypic plasticity of macrophages in our 3D co-culture model.

### 3D Macrophage-TNBC cancer cell co-culture induced major gene expression profile and functional enrichment changes in both cell types

**Fig 6:**
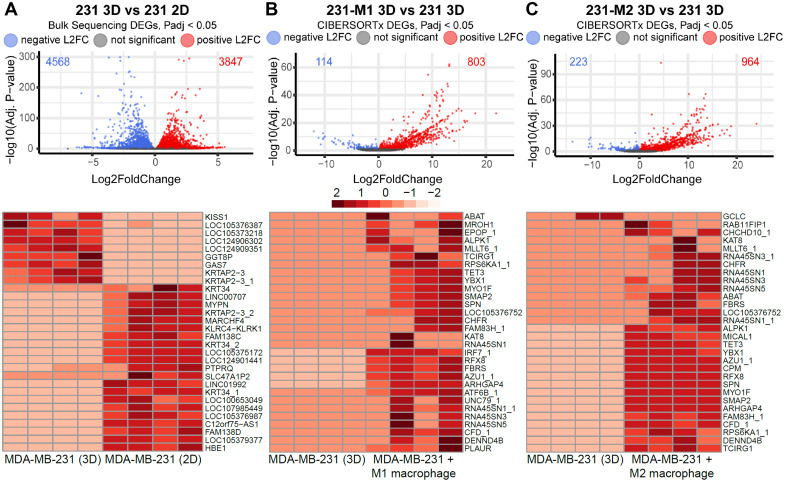
Differential gene expression analysis of MDA-MB-231 cells in 2D monolayer culture, 3D mono-cultured, and co-cultured spheroids with M1/M2-like macrophages. MDA-MB-231 cells were mono-cultured (A) or co-cultured with M1- (B) or M2-like (C) macrophages. Bulk sequencing results from co-cultured cells were processed using CIBERSORTx to separate different cell types by deconvolution analysis. Volcano plots of log2 fold changes showing the overall distribution of DEGs of MDA-MB-231 cells in different groups. Red (upregulated) and blue (downregulated) dots indicate significantly differing expression in genes (p-adj  <  0.05). The top 30 DEGs, based on absolute log2 fold change, from each comparison of MDA-MB-231 cells in different groups are displayed as row-scaled heatmaps. High expressions are indicated in red, and low expressions are indicated in white.

**Fig 7:**
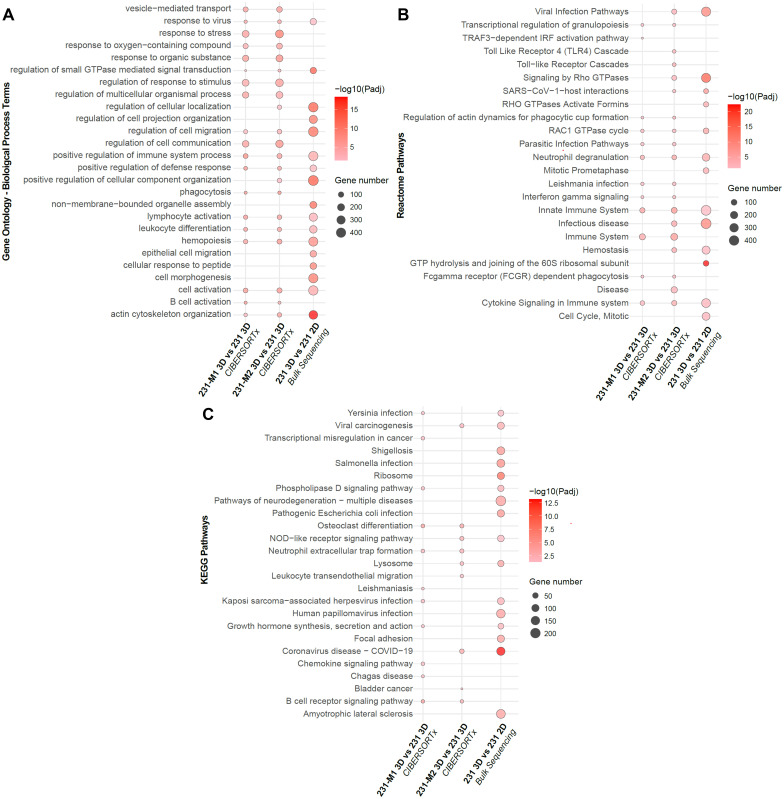
Enrichment analysis in MDA-MB-231 cells from 2D monolayer culture, 3D mono-cultured, and co-cultured spheroids with M1/M2-like macrophages. MDA-MB-231 cells were mono-cultured or co-cultured with M1- or M2-like macrophages. Bulk sequencing results from co-cultured cells were processed using CIBERSORTx to separate expression profiles of different cell types by deconvolution analysis. Lists of DEGs from each comparison were subject to enrichment analysis to identify overrepresented Gene Ontology – Biological Process (GO) Terms (A), Reactome pathways (B), and Kyoto Encyclopedia of Genes and Genomes (KEGG) Pathways (C) [27–34]. The resulting terms were clustered to reduce redundancy and provide a more comprehensive list of observed changes. Within the plot, dot size is used to indicate the number of member genes for each term that is included within observed DEGs, while dot color indicates significance, with red being more significant and white representing values closer to our threshold of adjusted p-value < 0.05.

To investigate the detailed influence of macrophage phenotypes on cancer cells, we analyzed the gene expression profiles, as well as the enrichment of GO, KEGG, and Reactome pathways, in MDA-MB-231 cells after mono-culturing or co-culturing with M1- or M2-like macrophages using RNA-seq. We identified 8,415 DEGs between 3D and 2D MDA-MB-231 cells, of which 3,847 were upregulated and 4,568 were downregulated, with DEGs including both tumor-promoting and tumor-suppressing genes (Fig 6 and S4, S32 Tables). These genes regulated by 3D formation were involved in various cellular functions and pathways, including cell migration, cell organization, cell morphogenesis, GTPases signaling, cell cycle, and TNF signaling (Fig 7). Complete enrichment results for Reactome, KEGG, and GO-BP are available in supplementary materials (S7, S10 and S13 Tables).

Co-culture with M1- or M2-like macrophages caused different gene expression profile changes with a small overlap of DEGs in the 3D MDA-MB-231 cells. CIBERSORTx high-resolution imputed gene counts from 3D cancer cells co-cultured with M1-like macrophages resulted in 803 upregulated and 114 downregulated DEGs when compared to 3D MDA-MB-231 cells in a mono-culture (Fig 6 and S5 Table). These DEGs were mainly involved in defense responses, immune system processing, cell communication, GTPase signaling, B-cell signaling, osteoclast differentiation, and chemokine signaling (Fig 7 and S8, S11, S14 Tables). In 3D MDA-MB-231 cells co-cultured with M2-like macrophages, 964 upregulated and 223 downregulated DEGs were observed (Fig 6 and S6 Table), which were involved in vesicle-mediated transport, response to stimulus, cell activation, viral infection pathway, GTPase signaling, osteoclast differentiation, and B cell signaling (Fig 7 and S9, S12, S15 Tables). The top 30 DEGs from each comparison are shown in the heatmap, and a brief overview of primary DEG functions is provided in S33 and S34 Tables.

**Fig 8:**
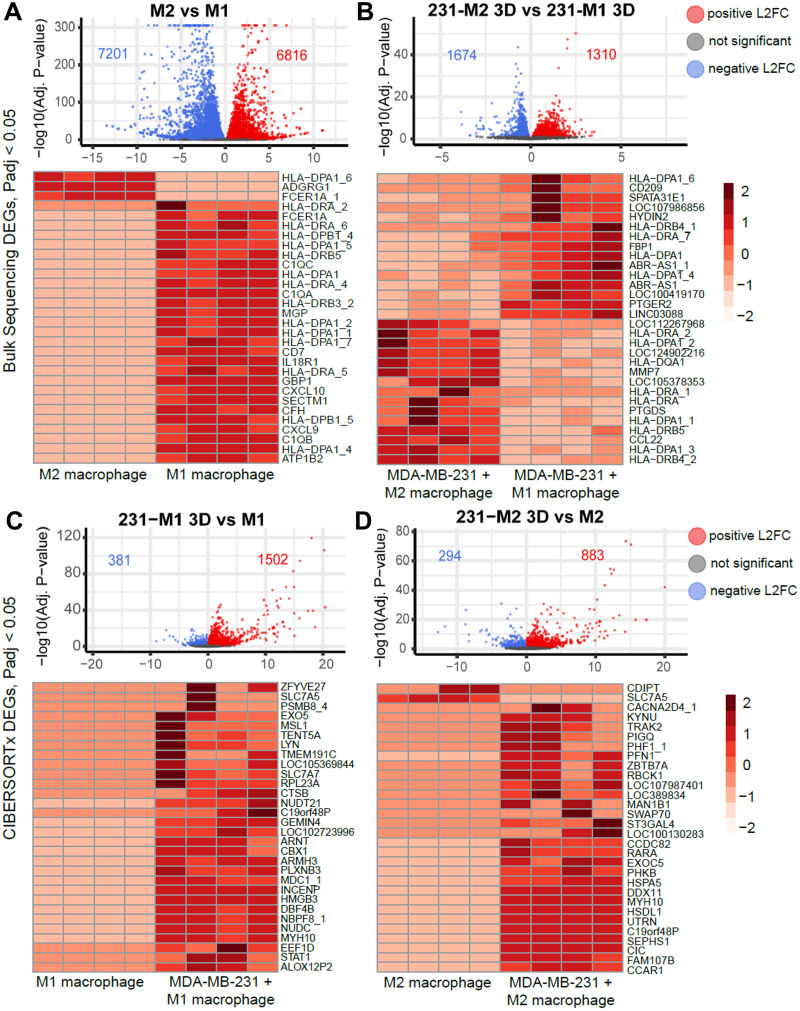
Differential gene expression analysis of M1 and M2-like macrophages in 2D monolayer culture and 3D co-cultured spheroids with MDA-MB-231 cells. Polarized M1/M2-like macrophages (A) and co-cultured MDA-MB-231 + M1/M2-like macrophages (B) were collected for RNA extraction and sequencing. Bulk sequencing results from co-cultured cells were processed using CIBERSORTx to separate different cell types by deconvolution analysis to identify DEGs attributed to M1 (C) or M2-like (D) macrophages within the co-cultured populations. Volcano plots of log2 fold changes showing the overall distribution of DEGs of M1/M2-like macrophages in different groups. Red (upregulated) and blue (downregulated) dots indicate significantly differing expression in genes (p-adj  <  0.05). The top 30 DEGs, based on absolute log2 fold change, from each comparison of macrophage-like cells in different groups are displayed as row-scaled heatmaps. High expressions are indicated in red, and low expressions are indicated in white.

**Fig 9:**
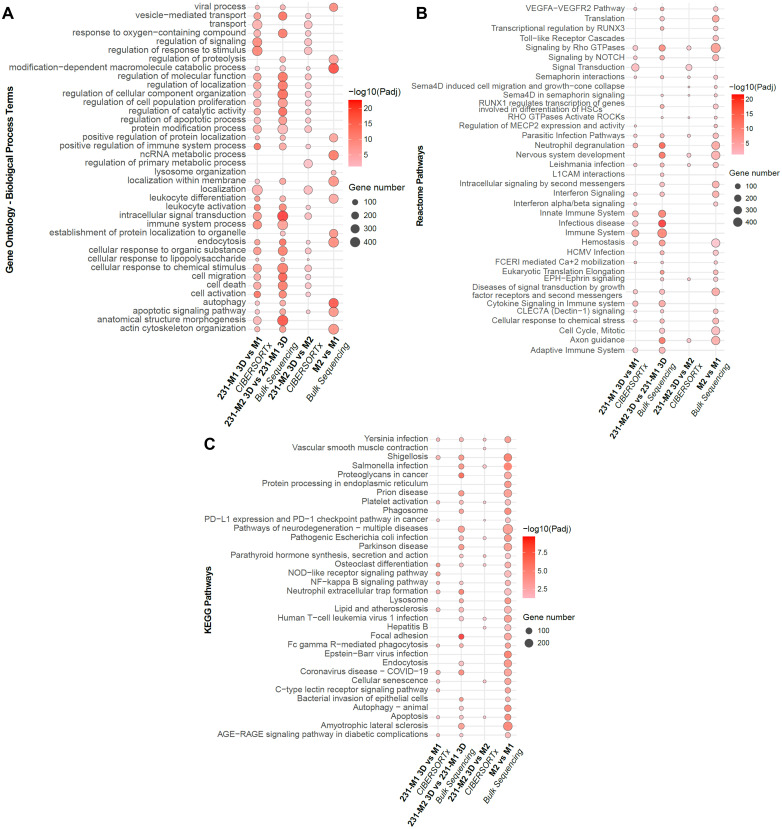
Enrichment Analysis in M1/M2-like macrophages from 2D monolayer culture and 3D co-cultured spheroids with MDA-MB-231 cells. Polarized M1/M2-like macrophages and co-cultured MDA-MB-231 + M1/M2-like macrophages were collected for RNA extraction and sequencing. Bulk sequencing results from co-cultured cells were processed using CIBERSORTx to separate different cell types by deconvolution analysis to identify DEGs attributed to M1 or M2-like macrophages within the co-cultured populations. Lists of DEGs from each comparison were subject to enrichment analysis to identify overrepresented Gene Ontology – Biological Process (GO) Terms (A), Reactome pathways (B), and Kyoto Encyclopedia of Genes and Genomes (KEGG) Pathways (C) [27–34]. Within the plot, dot size is used to indicate the number of member genes for each term that is included within observed differentially expressed genes, while dot color indicates significance, with red being more significant and white representing values closer to our threshold of adjusted p-value < 0.05.

We also compared gene expression profiles and functional enrichment among individually cultured M1/M2-like macrophages and co-cultured M1/M2-like macrophages in 3D spheroids with MDA-MB-231 cells. We found that co-culture with MDA-MB-231 cells significantly reduced the number of DEGs between M2- and M1-like macrophages (1310 upregulated and 1674 downregulated genes in co-culture; 6816 upregulated and 7201 downregulated genes in individual culture), supporting a likely phenotype transition as indicated by CIBERSORTx fractions between these two types of macrophages during the co-cultures (Fig 8 and S16, S17 Tables). The top DEGs between single cultured M1- and M2-like macrophages showed a successful polarization from THP-1 cells (Fig 8 and S35 Table). After co-culturing with MDA-MB-231 cells in 3D spheroids, more cancer response-related genes were found among the top DEGs instead of macrophage-subtype-related markers (Fig 8 and S36 Table). These genes were primarily involved in responses to oxygen-containing compounds, intracellular signal transduction, cell migration, morphogenesis of anatomical structures, nervous system development, infectious diseases, proteoglycans in cancer, and focal adhesion (Fig 9 and S20, S21, S24, S25, S28, S29 Tables).

When comparing the same macrophage type between co-cultures with MDA-MB-231 cells and individual cultures, 1883 DEGs (1502 upregulated and 381 downregulated) in M1-like macrophages and 1177 DEGs (883 upregulated and 294 downregulated) in M2-like macrophages were observed (Fig 8 and S18, S19 Tables). Both pro-inflammatory and anti-inflammatory genes were found within the top DEGs of co-cultured macrophages, indicating co-culturing with MDA-MB-231 cells in 3D spheroids induced a mixed M1- and M2-like phenotype in the macrophages (Fig 8 and S37, S38 Tables). DEGs in M1-like macrophages after co-culturing with 3D MDA-MB-231 cells were mainly enriched in the regulation of immune system processes, leukocyte activation, regulation of multicellular organismal processes, nervous system development, infectious disease, osteoclast differentiation, and the NOD-like receptor signaling pathway (Fig 9 and S22, S26, S30 Tables). The DEGs in M2-like macrophages were mainly enriched in intracellular signal transduction, establishment of localization, regulation of molecular function, the RHO GTPase cycle, mitotic cell cycle, parathyroid hormone synthesis, and cellular senescence (Fig 9 and S23, S27, S31 Tables). In summary, our RNA-seq analysis revealed significant transcriptome-wide changes in both cancer cells and macrophages in the 3D co-cultures. Functional enrichment analysis indicated that these genes were involved in multiple pathways, such as immune response, metabolic regulation, and defense response. These findings provided a robust molecular foundation for understanding the mechanisms of interaction between macrophages and TNBC cancer cells and highlighted potential targets for future therapeutic interventions.

Cytokine secretion levels in the medium of MDA-MB-231 cells 2D culture, 3D monoculture, and co-cultures with M1/M2 macrophages were also analyzed. We found that compared to 2D culture, IL-6, IL-8 and VEGF levels were significantly increased in 3D culture. Both M1 and M2 co-cultures showed significantly elevated IL-10, IL-12p70 and RANTES levels, while only M1 co-culture showed increased IL1α, IL1β, IL-8, GRO, MCP-1, MIP-1α, MIP-1β and MMP-9 levels (S7 Fig).

## Discussion

*In vitro* 3D spheroids are typically considered a more physiologically relevant model than 2D cell cultures in cancer research related to therapeutic development, due to their ability to recapitulate the complex tumor microenvironment (TME). However, a challenging limitation in current studies of 3D models is often the lack of immune cells, which play crucial roles in regulating both protumorigenic and antitumorigenic responses during cancer development [37]. In our study, we established a co-culture 3D spheroid model of differentiated M1- or M2-like macrophages derived from THP-1 cells and MDA-MB-231 cells to investigate the bi-directional interactions between macrophages and cancer cells rather than focusing solely on the changes of a single cell type.

One major and interesting finding in our study is that, rather than maintaining their polarization status, M1- and M2-like macrophages partially transitioned their phenotypes to resemble each other after 4 days of co-culture with MDA-MB-231 cells in 3D spheroids, resulting in a mixed M1/M2 macrophage population in the co-cultures. The RNAseq analysis showing a sharply decreased number of DEGs between M1 and M2-like macrophages after co-culture with cancer cells indicated a transcriptional convergence in the co-cultured macrophages. Combined with the co-existence of both pro-inflammatory and anti-inflammatory genes found within the co-cultured macrophages, our results supported the transition plasticity of macrophage phenotypes in the co-cultures. In cancer development, M1 macrophages are believed to express high levels of pro-inflammatory cytokines and reactive nitrogen and oxygen intermediates, which lead to tumor growth inhibition [38,39]. In contrast, M2 macrophages promote tumor cell proliferation, migration, and invasion by secreting anti-inflammatory cytokines and angiogenic factors [40]. However, TAMs tend not to adhere to one subtype and change states in the development of cancer due to their high plasticity. Stewart et al. found that basal-like breast cancer cells induced widespread phenotypic changes associated with both M1 and M2 macrophage polarization [41]. Another *in vitro* study also observed that TNBC-conditioned media could induce macrophage polarization with a mixture of M1 and M2 phenotypes [42]. In our study, besides the mixed macrophage phenotype markers expression and mixed inflammatory genes expression, increased pro-inflammatory (IL-12p70) and anti-inflammatory (IL-10) cytokine levels were also found in both M1 and M2 macrophages and MDA-MB-231 co-cultures, which could also support our hypothesis and are consistent with previous research.

Cell-type deconvolution of our bulk transcriptomic data also showed a shifting trend in cell proportions between M1- and M2-like macrophages in 3D co-cultured spheroids with MDA-MB-231 cells, supporting our observation of phenotypic plasticity in these co-cultured macrophages described above. Interestingly, we found that the fraction of M1-like macrophages shifting to an M2-like phenotype, approximately 10%, was much lower than the fraction of M2-like macrophages shifting to an M1 phenotype, approximately 40%, in the 3D co-cultures. This difference could be related to the time point at which we added macrophages to the 3D MDA-MB-231 cell spheroids and sequenced the RNA 4 days after co-culture. Steenbrugge et al. found that TAMs showed an M1-type phenotype in the early stages of co-inoculation of macrophages and TNBC cells in a mouse model, while an M2-type phenotype appeared later [43]. In a recent study, it was recommended to use the TAM balance fraction, MBF, the fraction of M2-TAMs to M1-TAMs, to assess the status of TAMs in the TME, as a high MBF was related to poor prognosis and tumor metastasis in breast cancer development [44]. This ratiometric approach could be a valuable tool for evaluating dynamic changes in the tumor environment, as we also observed that even though macrophage phenotype shifting occurred in the co-cultured tumor spheroids, the influence of M1- or M2-like macrophages on the cancer cells still differed significantly. This was also supported by our observation that increased pro-inflammatory cytokine levels of IL-1, GRO, CCL3, and CCL4 were observed only in the M1 macrophage and MDA-MB-231 co-cultures compared with MDA-MB-231 monocultures. One of the current popular macrophage-targeted therapies is TAM reprogramming, which focuses on shifting M2-like macrophages into more anti-tumorigenic, inflammatory M1-like phenotypes using nanotechnologies [14]. Our 3D co-culture system containing the dynamic changes of macrophage phenotypes could be a useful tool for monitoring the effects of this treatment strategy on the fractions of macrophage shifting.

Beyond macrophage phenotype dynamics, the 3D co-culture system itself introduced significant changes in cancer cell behavior compared to conventional 2D cultures. Multiple studies have demonstrated that cancer cells cultured in 3D spheroids exhibit distinct differences in gene expression, protein markers, cell receptors, and related pathways compared to those cultured in 2D [45,46]. In our study, MDA-MB-231 cells in 3D spheroids exhibited lower viability and a slower proliferation rate compared to 2D monolayer culture, as well as increased chemoresistance to doxorubicin and paclitaxel treatment. Additionally, numerous differentially expressed genes and various involved pathways were identified, which is consistent with previous studies [47,48]. The spontaneously increased levels of IL-6, IL-8, and VEGF in our MDA-MB-231 3D spheroids suggest a possible mesenchymal, stem-cell-like phenotype in the 3D cancer cells when compared with 2D monolayer culture, and could better recapitulate the hyper-inflammatory, pro-angiogenic *in vivo* TME. Our confocal images of 3D MDA-MB-231 spheroids showed that both doxorubicin and paclitaxel could diffuse into the core of the spheroids, with dead cells distributed relatively evenly. This indicates that drug diffusion is likely not the primary reason responsible for chemoresistance in 3D cancer spheroids.

Both our monoculture and co-culture 3D systems in the ultralow attachment (ULA) u-bottom plates produced relatively spherical, compact structures that maintained their shapes when the culture medium was changed or when transferred to glass-bottom dishes for washing and imaging. Some studies have reported that the addition of Matrigel or other viscosity-raising compounds are required to obtain more compact spheroids of MDA-MB-231 cells, due to their low E-cadherin expression [49, 50]. However, our study produced similar results using the ultra-low attachment plate without adding those reagents, consistent with other research using similar methods [51–53], even though the outline was not as perfectly well-defined as those with extracellular matrix added. The Nunclon Sphera surface of the ULA is coated with a hydrophilic polymer, which supports the formation of consistent cancer spheroids in suspension. This kind of spheroid formation could still be explained by intercellular adhesions in MDA-MB-231 cells, but by collagen I/integrin β1 interactions rather than E-cadherin, according to previous research [53, 54]. However, due to various cultural conditions, including culture plate types, medium supplementation, coating conditions, and cell seeding densities, the formation and characteristics of 3D spheroids can vary significantly. In our study, with a diameter of around 900 µm and a depth of around 360 µm, our formed 3D models were more of an ellipsoid shape, which was consistent with other research using the same methods [53]. In addition to the ellipsoid shape, due to the intercellular adhesion characteristic of MDA-MB-231 cells and the lack of additional extracellular matrix supplementation, the inner structure of our 3D systems might not be as compact, which could explain why the chemotherapy drugs could enter the core of our spheroids without an obvious necrotic core being observed.

Among the phenotypic changes associated with 3D culture, EMT is particularly relevant to TNBC aggressiveness. Epithelial-to-mesenchymal transition (EMT) is an important process occurring in TNBC related to tumor aggressiveness, metastasis, and chemoresistance [18]. In our study, we observed upregulated levels of N-cadherin, fibronectin, α-SMA, and FSP1, and downregulated laminin in 3D mono-cultured MDA-MB-231 cells compared with 2D culture, suggesting that the cancer cells underwent an EMT in the 3D spheroids and might acquire an aggressive malignant phenotype. The observed decrease in E-cadherin and vimentin, and an increase in β-catenin 1 MFI, but not cell counts, in 3D MDA-MB-231 cultures compared with 2D cultures, indicates altered protein expression per cell without affecting overall cell viability and population count. MDA-MB-231 cells are known to exhibit mesenchymal characteristics and low E-cadherin expression. The decrease in E-cadherin and vimentin, and increase in β-catenin 1 density per cell in 3D spheroids also support the EMT induction in the 3D spheroids. Studies have shown that increased cell-cell connections, nutrient gradients, and structural constraints involved in spheroid formation might contribute to the EMT transition and chemoresistance in 3D cancer models [18,48]. Interestingly, co-culture with either M1- or M2-like macrophages partly attenuated the EMT process in MDA-MB-231 cells in 3D spheroids, while increased cancer cell viability but not proliferation rate was only observed in 3D co-cultures with M2-like macrophages. This could be due to the different degrees of macrophage phenotype shifting in the co-culture spheroids as mentioned above, and indicates that macrophages may regulate tumor growth by modifying other environmental factors rather than directly stimulating cancer cell division [55]. In addition, increased IL12p70 levels were observed in both M1 and M2 macrophage co-cultured 3D models, which could also be related to the increased drug response in 3D co-cultures, due to its ability to shift the macrophage secretome and block the tumor-supportive crosstalk cascade pathways such as STAT3 [55].

Closely linked to EMT is the cancer stem cell phenotype, which we assessed through CD44 and CD24 expression. Studies have reported that CD44-positive cells exhibit a more mesenchymal-like profile and are involved in the proliferation and angiogenesis of cancer cells, while CD24-positive cells are more closely related to metastatic potential and poor clinical outcomes in breast cancer [56]. CD44 + /CD24- cancer cells are believed to represent cancer stem cells and show high expression in TNBC cells [57,58]. We also observed high expression of CD44 + /CD24- subtype cells in all groups of MDA-MB-231 cells. Interestingly, expression of both CD44 + /CD24+ and CD44-/CD24 + significantly increased in MDA-MB-231 cells in 3D spheroids, with only M2-like macrophages further upregulating these two subtypes in the 3D co-cultures. Although both tumor-promoting and tumor-suppressing DEGs existed in our co-cultured 3D spheroids, more positive response immune pathways were enriched in the M1-like macrophage co-cultures compared with M2-like macrophages. These findings indicated that even though there is a phenotype shift between M1- and M2-like macrophages in our 3D co-culture model with MDA-MB-231 cells, the involved genes and related pathways showed a different trend, with M1-like macrophages still showing a relative anti-tumor effect.

Although our 3D co-culture model spheroids contained macrophages and TNBC cancer cells, some limitations remain due to the complexity of the TME. Multiple cell types are involved in the development of the cancer environment. Therefore, integrating additional cell types, including other immune cells and endothelial cells, into cancer spheroids should be considered in subsequent studies. Considering the dynamic changes in macrophage polarization and their influence on cancer cells, multiple time-point analysis could further monitor the transition process. In addition, validation across different TNBC cell lines and multiple drug concentrations in the 3D co-culture system should be considered when evaluating the efficacy of novel cancer therapies. While our transcriptomic analysis provided a valuable and robust foundational overview of interactions between co-cultured macrophages and cancer cells, a limitation of lacking orthogonal experimental validation still exists. In particular, our cell fractions were estimated using CIBERSORTx deconvolution based on transcriptomic similarity to individually cultured reference profiles, whose accuracy may be potentially affected by co-culture-induced transcriptional drift, differences in cellular mRNA content, or technical variation. Further validation using single-cell RNA sequencing or functional evidence for key genes and pathways would strengthen these findings, uncover the mechanisms involved, and lay the foundation for future clinical applications.

In conclusion, our 3D co-culture model of MDA-MB-231 cells and polarized macrophages recapitulated aspects of the complex environment in tumor development and revealed important dynamic interactions between macrophages and tumor cells, laying the foundation for establishing a more complex 3D *in vitro* co-culture system. The transition between two macrophage types when co-cultured with cancer cells in spheroids and their distinct influences on tumor development and treatment response suggest that combining chemotherapies with immunotherapies targeting TAM phenotype transitions could be a potential and effective strategy to improve TNBC outcomes.

## Supporting information

S1 Fig Establishment of 3D co-culture spheroids of MDA-MB-231 cells and M1/M2-like polarized macrophages. THP-1 cells were stimulated with 100 nM PMA for 24 h, followed by treatment with 50ng/mL IFN-γ or 50ng/mL IL-4 for 24 h. Polarized macrophages were washed to remove polarizing cytokines and seeded into each well containing MDA-MB-231 cells for co-culture and incubated for an additional 4 days before further treatment or analysis. 50 μM DOX or PTX was added to the cells for an additional 48 h for treatment studies. Created in BioRender. Combs, C. (2025) https://BioRender.com/o00c027.
(TIF)

S2 Fig Chemosensitivity of MDA-MB-231 cell monocultures or co-culture with M1/M2 macrophages in a 2D monolayer. (A-D) 2D or 3D monocultured MDA-MB-231 cells were exposed to different concentrations of paclitaxel (A, B) or doxorubicin (C, D) for 48h. (E-F) MDA-MB-231 and M1/M2 macrophages 2D co-cultures were exposed to 50 μM paclitaxel or doxorubicin for 48h. WST assays were performed to assess cell viability. Results from the treated groups were compared with the vehicle group. Results are presented as mean ± SD; n = 7, p* < 0.05, p** < 0.01, p*** < 0.001, p**** < 0.0001.
(TIF)

S3 Fig Gating strategy of a representative 3D co-culture sample. MDA-MB-231 cells and M1/M2-like macrophages from 2D monolayer, 3D mono-cultured, and co-cultured spheroids were stained using the previously described antibodies for quantitative analysis. (A) Samples were gated on live cells using SSC-A/FSC-A gating. (B) Debris and doublets were excluded from FSC-A/FSC-H gating. (C) Live cells were selected from side scatter gating. (D) Representative sample of separating cancer cells (CD45-) and macrophages (CD45+) for sequential analysis.
(TIF)

S4 Fig Representative images of 3D reconstruction from Z-stack confocal images of co-cultured spheroids. Pre-stained MDA-MB-231 cells (green) were co-cultured with (A) M1 (red) or (B) M2 (purple) macrophages for 4 days and observed under a confocal microscope. (C) Quantification of macrophage infiltration percentage in the spheroids based on Z-stack images. Results are presented as mean ± SD; n = 3. p* < 0.05, p** < 0.01, p*** < 0.001, p**** < 0.0001.
(TIF)

S5 Fig Representative flow cytometry histograms/plots (A) and median fluorescence intensity (MFI) (B) of MDA-MB-231 cells phenotype changes. MDA-MB-231 cells were mono-cultured or co-cultured with M1- or M2-like macrophages for 4 days. Results are presented as mean ± SD; n = 3, p* < 0.05, p** < 0.01, p*** < 0.001, p**** < 0.0001.
(TIF)

S6 Fig Representative flow cytometry histograms (A) and median fluorescence intensity (MFI) (B) of M1/M2 macrophages phenotype changes. M1 and M2-like macrophages were collected after polarization or after co-culturing with MDA-MB-231 cells in 3D spheroids for 4 days for flow cytometry analysis. Results are presented as mean ± SD; n = 3, p* < 0.05, p** < 0.01, p*** < 0.001, p**** < 0.0001.
(TIF)

S7 Fig Cytokine levels in the medium of MDA-MB-231 2D monolayer culture, 3D mono-culture, and co-culture spheroids with M1/M2-like macrophages. MDA-MB-231 cells were mono-cultured or co-cultured with M1- or M2-like macrophages for 4 days and media were collected for cytokine array analysis. Results are presented as mean ± SD; n = 4, p* < 0.05, p** < 0.01, p*** < 0.001, p**** < 0.0001.
(TIF)

S1 Table Antibody Information. (XLSX)

S2 Table Cell Viability Data of Ghost Dye Analysis. (XLSX)

S3 Table Percentages of Macrophage Cells Expressing Different Markers (Flow Cytometry Analysis). (XLSX)

S4 Table Differentially Expressed Genes between 231 3D vs 231 2D cells. (XLSX)

S5 Table Differentially Expressed Genes between CIBERSORTx imputed counts for 231 3D cells co-cultured with M1 cells vs 231 3D cells. (XLSX)

S6 Table Differentially Expressed Genes between CIBERSORTx imputed counts for 231 3D cells co-cultured with M2 cells vs 231 3D cells. (XLSX)

S7 Table Gene Ontology Biological Process Enrichment Clusters for 231 3D vs 231 2D DEGs. (XLSX)

S8 Table Gene Ontology Biological Process Enrichment Clusters for DEGs between CIBERSORTx imputed counts for 231 3D cells co-cultured with M1 cells vs 231 3D cells. (XLSX)

S9 Table Gene Ontology Biological Process Enrichment Clusters for DEGs between CIBERSORTx imputed counts for 231 3D cells co-cultured with M2 cells vs 231 3D cells. (XLSX)

S10 Table Kyoto Encyclopedia of Genes and Genomes Enrichment Terms for 231 3D vs 231 2D DEGs. (XLSX)

S11 Table Kyoto Encyclopedia of Genes and Genomes Enrichment Terms for DEGs between CIBERSORTx imputed counts for 231 3D cells co-cultured with M1 cells vs 231 3D cells. (XLSX)

S12 Table Kyoto Encyclopedia of Genes and Genomes Enrichment Terms for DEGs between CIBERSORTx imputed counts for 231 3D cells co-cultured with M2 cells vs 231 3D cells. (XLSX)

S13 Table Reactome Pathways Enrichment Results for 231 3D vs 231 2D DEGs. (XLSX)

S14 Table Reactome Pathways Enrichment Results for DEGs between CIBERSORTx imputed counts for 231 3D cells co-cultured with M1 cells vs 231 3D cells. (XLSX)

S15 Table Reactome Pathways Enrichment Results for DEGs between CIBERSORTx imputed counts for 231 3D cells co-cultured with M2 cells vs 231 3D cells. (XLSX)

S16 Table Differentially Expressed Genes between M1 vs M2 cells. (XLSX)

S17 Table Differentially Expressed Genes between 231 3D + M2 vs 231 3D + M1 co-cultures. (XLSX)

S18 Table Differentially Expressed Genes between CIBERSORTx imputed counts for M1 cells co-cultured with 231 3D cells vs M1 cells. (XLSX)

S19 Table Differentially Expressed Genes between CIBERSORTx imputed counts for M2 cells co-cultured with 231 3D cells vs M2 cells. (XLSX)

S20 Table Gene Ontology Biological Process Enrichment Clusters for M2 vs M1 DEGs. (XLSX)

S21 Table Gene Ontology Biological Process Enrichment Clusters for 231 3D + M2 vs 231 3D + M1 DEGs. (XLSX)

S22 Table Gene Ontology Biological Process Enrichment Clusters for DEGs between CIBERSORTx imputed counts for M1 cells co-cultured with 231 3D cells vs M1 cells. (XLSX)

S23 Table Gene Ontology Biological Process Enrichment Clusters for DEGs between CIBERSORTx imputed counts for M2 cells co-cultured with 231 3D cells vs M2 cells. (XLSX)

S24 Table Kyoto Encyclopedia of Genes and Genomes Enrichment Terms for M2 vs M1 DEGs. (XLSX)

S25 Table Kyoto Encyclopedia of Genes and Genomes Enrichment Terms for 231 3D + M2 vs 231 3D + M1 DEGs. (XLSX)

S26 Table Kyoto Encyclopedia of Genes and Genomes Enrichment Terms for DEGs between CIBERSORTx imputed counts for M1 cells co-cultured with 231 3D cells vs M1 cells. (XLSX)

S27 Table Kyoto Encyclopedia of Genes and Genomes Enrichment Terms for DEGs between CIBERSORTx imputed counts for M2 cells co-cultured with 231 3D cells vs M2 cells. (XLSX)

S28 Table Reactome Pathways Enrichment Results for M2 vs M1 DEGs. (XLSX)

S29 Table Reactome Pathways Enrichment Results for 231 3D + M2 vs 231 3D + M1 DEGs. (XLSX)

S30 Table Reactome Pathways Enrichment Results for DEGs between CIBERSORTx imputed counts for M1 cells co-cultured with 231 3D cells vs M1 cells. (XLSX)

S31 Table Reactome Pathways Enrichment Results for DEGs between CIBERSORTx imputed counts for M2 cells co-cultured with 231 3D cells vs M2 cells. (XLSX)

S32 Table Function of Top DEGs between MDA-MB-231 3D and MDA-MB-231 2D. (XLSX)

S33 Table Function of Top DEGs between MDA-MB-231 + M1 3D and MDA-MB-231 3D. (XLSX)

S34 Table Function of Top DEGs between MDA-MB-231 + M2 3D and MDA-MB-231 3D. (XLSX)

S35 Table Function of Top DEGs between polarized M2 and M1. (XLSX)

S36 Table Function of Top DEGs between MDA-MB-231 + M2 3D and MDA-MB-231 + M1 3D. (XLSX)

S37 Table Function of Top DEGs between MDA-MB-231 + M1 3D and polarized M1. (XLSX)

S38 Table Function of Top DEGs between MDA-MB-231 + M2 3D and polarized M2. (XLSX)
